# Immunotherapy Summary for Cytokine Storm in COVID-19

**DOI:** 10.3389/fphar.2021.731847

**Published:** 2021-09-17

**Authors:** Yaqun Li, Wenjie Zhao, Jinhua Liu, Zichao Chen, Qingtao Lv, Zhen Zhang

**Affiliations:** ^1^ Innovation Research Institute of Traditional Chinese Medicine, Shandong University of Traditional Chinese Medicine, Jinan, China; ^2^ College of Pharmacy, Shandong University of Traditional Chinese Medicine, Jinan, China; ^3^ Experimental Center, Shandong University of Traditional Chinese Medicine, Jinan, China

**Keywords:** immunotherapy, cytokine storm, COVID-19, cytokine inhibitors, SARS-CoV-2

## Abstract

COVID-19 pneumonia caused by severe acute respiratory syndrome coronavirus 2 (SARS-CoV-2) has ravaged the world, resulting in an alarming number of infections and deaths, and the number continues to increase. The pathogenesis caused by the novel coronavirus was found to be a disruption of the pro-inflammatory/anti-inflammatory response. Due to the lack of effective treatments, different strategies and treatment methods are still being researched, with the use of vaccines to make the body immune becoming the most effective means of prevention. Antiviral drugs and respiratory support are often used clinically as needed, but are not yet sufficient to alleviate the cytokine storm (CS) and systemic inflammatory response syndrome. How to neutralize the cytokine storm and inhibit excessive immune cell activation becomes the key to treating neocoronavirus pneumonia. Immunotherapy through the application of hormones and monoclonal antibodies can alleviate the immune imbalance, but the clinical effectiveness and side effects remain controversial. This article reviews the pathogenesis of neocoronavirus pneumonia and discusses the immunomodulatory therapies currently applied to COVID-19. We aim to give some conceptual thought to the prevention and immunotherapy of neocoronavirus pneumonia.

## Introduction

Since the end of 2019, novel coronavirus has been gradually spread worldwide, which not only poses a severe challenge to the global public health system but also brings huge economic losses to the society. Virus infection can cause cytokine storm, and then lead to acute respiratory distress syndrome (ARDS), and even multiple organ failure until death (Zhu et al., 2020).

Many acute infections have a strong and potentially destructive effect on the immune response. Of course, this immune response can reduce self-harm to a certain extent (Cavalli and Dagna et al., 2020). Once the infection develops to the advanced stage, patients often have severe disease symptoms. If the immune cascade cannot be properly controlled, the immune response may have adverse effects on the host, and remodeling the balance between inflammation and anti-inflammation may be a more effective method for the treatment of infection (Puelles et al., 2020). This paper focuses on the mechanism of CS induced by the SARS-CoV-2 virus and its clinical manifestations, and reviews the recently reported immunotherapies for cytokine storm, with the expectation of providing a reference for the treatment of COVID-19.

## Immunopathogenesis of Cytokine Storm

**FIGURE 1:**
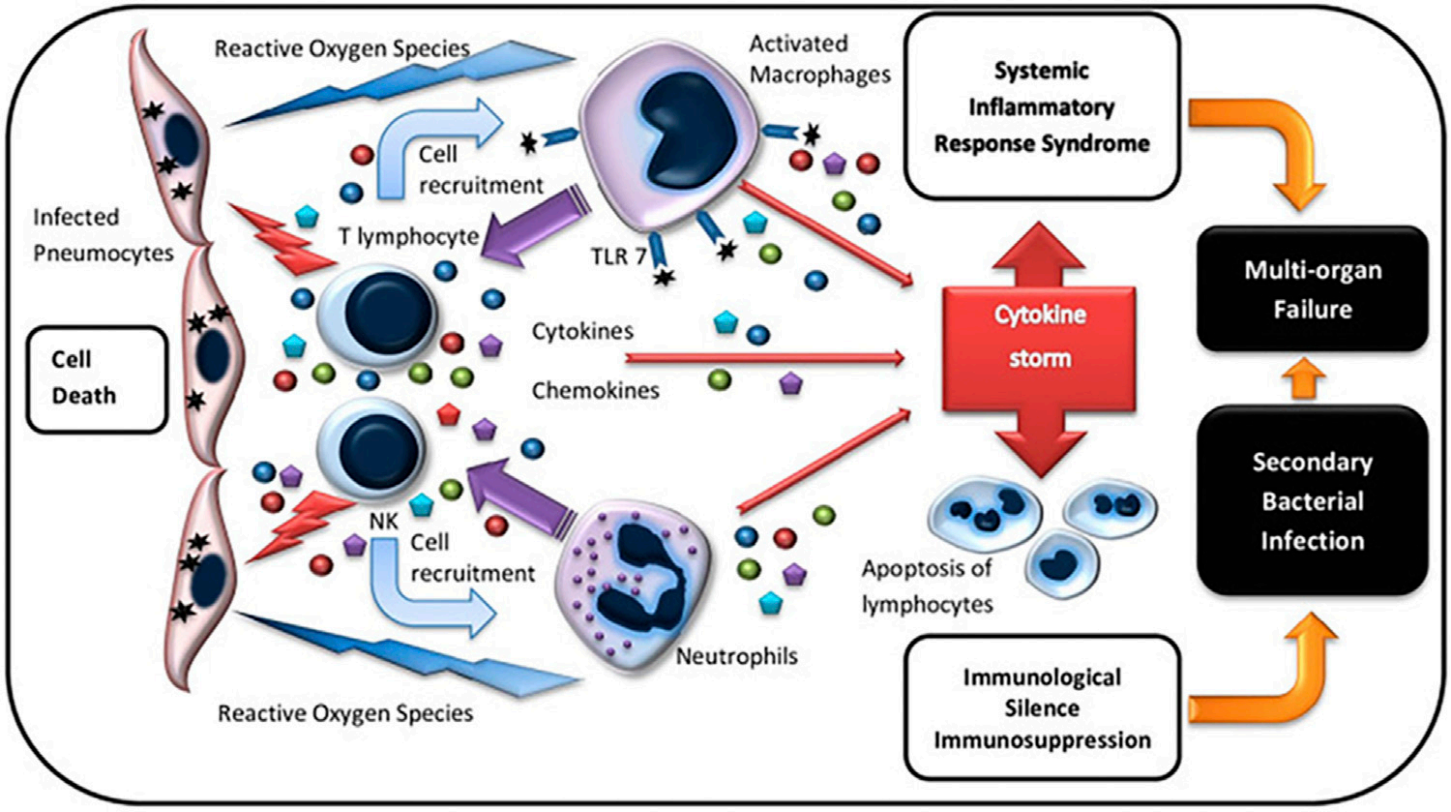
| Immunopathogenesis of cytokine storm (Kiselevskiy et al., 2020).

The potential source of CS is the megakaryocyte and some subsets of monocyte indicated by sequencing data of single-cell transcriptome (Ren et al., 2021). After the human body is infected with SARS-CoV-2, innate immune cells such as neutrophils and natural killer (NK) cells (Hashimoto et al., 2013)are rapidly activated to secrete a variety of cytokines and chemokines, including IL-2, IL-10, TNF-α, IFN-γ, CXCL10, CCL2, CCL3, etc., And then activate the inflammatory cytokines storm (Huang et al., 2020; Wan et al., 2020; Chen et al., 2020). These explosions of cytokines and chemokines then activate cells of the adaptive immune system. T cells, which dominate the adaptive immune cells, differentiate into subsets with different effector cell functions and participate in CS (Figure 1) (Kiselevskiy et al., 2020).

COVID-19 enters cells through the angiotensin-converting enzyme 2 (ACE2) receptor, and cells that highly express ACE2 become the main targets of COVID-19 invasion (Zhou et al., 2020). What’s more, in the autopsy of the patients, the SARS-CoV-2 virus was found in the heart, liver, and other tissue samples (Varadhana et al., 2020), indicating that many organs were damaged (Synowiec et al., 2021).

The main clinical features of COVID-19 include fever, dyspnea, myalgia, dry cough, etc., (Lupia et al., 202; Grasselli et al., 2020; Guan et al., 2020). Atypical clinical features include digestive system symptoms (Mao et al., 2020), abnormal liver function (Yadav et al., 2021), dysfunction of smell or taste (Luers et al., 2020; Liou et al., 2021), insomnia (Spinato et al., 2020), etc., As the body responds to acute systemic inflammation, pro-inflammatory cytokines can also affect the brain and induce behavioral and physical symptoms, such as fever, nausea, and anorexia (Dantzer et al., 2008).

## Cytokine Storms in Severe COVID-19

As we all know, the cellular structure of CS is based on immune cells such as neutrophils and lymphocytes (Yang et al., 2018). Remarkably, hypolymphocyticemia is frequently found in COVID-19. It is reported that about 63% of patients have peripheral blood lymphocyte counts less than 1.0 × 109/L, and this proportion can be as high as 85% in critically ill patients (Huang et al., 2020). In a retrospective study involving 452 COVID-19 patients, the authors found a decrease in the lymphocytes, monocytes, also the ratio of eosinophils and basophils. Analysis of the lymphocyte subsets revealed that the severity of the disease corresponded to decreased number of B cells, T cells, and NK cells in COVID-19 patients (Qin et al., 2020). In addition, A study on pathological dissection of lymph nodes and spleen found that the number of B lymphocytes in lymph nodes decreased significantly. These phenomena also explain that humoral immune suppression is the reason for the rapid development of some severe patients’ illnesses (Kaneko et al., 2020).

A study on the immunological characteristics of peripheral blood showed that 4–6 days after the patient was infected with SARS-CoV-2, the number of T cells dropped to a minimum; on the contrary, the number of multiple cytokines reaches the highest level. These phenomena indicate that the decrease of T cells in the peripheral blood can lead to aggravation of the inflammatory response (Liu et al., 2020). One manifestation of CS caused by the massive production of cytokines is the hyperactivation of immune cells. Moreover, a novel coronavirus pneumonia pathology report shows that Interstitial mononuclear inflammatory infiltrates in both lungs. In the peripheral blood, the number of CD4 + and CD8 + T cells decreased significantly but excessively activated. Therefore, this team believes that these manifestations of excessive activation of T cells may be part of the causes of severe immunopathological damage to the lungs (Xu et al., 2020).

## Immunotherapy

### Cytokine Inhibitors

Targeting certain key cytokines in CS, using their monoclonal antibodies, and recombinant proteins, etc., to antagonize them and block their pro-inflammatory effects, is an effective strategy to treat cytokine storms.

### Inhibition of IL-6 Signaling

IL-6 mediates intercellular signal transduction, regulates immune cells, and has a strong pro-inflammatory effect (Taniguchi et al., 2020). The level of IL-6 in non-survivors is higher than that in survivors of COVID-19, indicating that increased levels of IL-6 in peripheral blood are closely related to worsening of the disease and poor prognosis (Ruan et al., 2020). Pathogenic T cells and monocytes can trigger inflammatory storms with a large number of interleukin-6 (Fu et al., 2020). Therefore, the use of monoclonal antibodies binding to the IL-6 receptor may quell the inflammatory storm.

Due to the overactive immune response in patients with severe COVID-19, the potential of tocilizumab in the treatment has been evaluated in many clinical trials. However, the results of previous clinical trials were inconsistent. In four randomized clinical trials (Salama et al., 2020; Stone et al., 2020; Hermine et al., 2021; Rosas et al., 2021), a single intravenous injection of tocilizumab (8 mg/kg) did not significantly improve mortality in hospitalized patients. In three retrospective clinical studies (Martínez-Sanz et al., 2020; Gupta et al., 2021; Horby et al., 2021), tocilizumab can reduce mortality. Especially for patients with high C-reactive protein (CRP) levels and systemic inflammation, tocilizumab can reduce the mortality rate and shorten the discharge time (Martínez-Sanz et al., 2020；; Horby et al., 2021; Cavalli et al., 2021).

### Inhibition of IL-1 Signaling

The IL-1 family is not to be ignored in CS, especially IL-1β (Shimabukuro-Vornhagen et al., 2018; Vardhana et al., 2020). Anakinra can simultaneously block the pro-inflammatory signals induced by IL-1a and IL-1b. It is widely used in the treatment of rheumatoid arthritis and protein-associated periodic fever syndrome (Quartier et al., 2010; Cavalli et al., 2015; Ben-Zvi et al., 2017). Animal experiments have shown that Anakinra can reduce the mortality of CRS patients caused by CAR-T therapy, and also reduces neurotoxicity due to its ability to cross the blood-brain barrier (Giavridis et al., 2018). Compared with other cytokine blockers such as tocilizumab, anakinra will become an excellent candidate for clinical trials of COVID-19 ARDS due to its good tolerability, low side effects, and short half-life. In addition, Canakinumab is a monoclonal antibody that selectively blocks IL-1b-induced pro-inflammatory signals, which can improve respiratory function and rapidly reduce systemic the inflammatory response (Sheng et al., 2020; Ucciferri et al., 2020).

Anakinra can improve the survival rate of non-invasive ventilation and had no significant adverse effects (Huet et al., 2020; Franzetti et al., 2021). A retrospective cohort study showed that patients who received high-dose of anakinra intravenously (5 mg/kg, twice a day) could choose to remove mechanical ventilation since their respiratory function was improved (Cavalli et al., 2020). Four other cohort studies (Franzetti et al., 2020; Huet et al., 2020; Iglesias-Julián et al., 2020; Pasin et al., 2021) similarly showed the beneficial efficacy of anakinra against COVID-19. However, further studies are needed to determine the appropriate timing and dosage of anakinra, as well as to screen for molecular classifications of individual inflammatory responses to ensure the maximum therapeutic effect. It is worth noting that because the use of glucocorticoids can lead to an increase in insulin demand, patients with type 2 diabetes should choose IL-1 inhibitors and use glucocorticoids with caution (Cavalli et al., 2021).

### Inhibition of GMC-SF Signaling

GMC-SF is a proinflammatory cytokine. In the early stage of SARS-CoV-2 infection, GM-CSF can enhance the immunity of the body and protect the lungs from secondary bacterial infection after the viral invasion (Bonaventura et al., 2020). However, in COVID-19 cases accompanied by a cytokine storm, the myeloid cell growth factor GM-CSF was significantly increased, possibly leading to the recruitment and activation of monocytes (Thwaites et al., 2021). In this case, too much GM-CSF is not helpful. Blocking GM-CSF signaling by binding to GM-CSF or GM-CSF receptors may help to ameliorate lung damage caused by excessive inflammation. In a retrospective cohort study to evaluate the efficacy of lenzilumab, the clinical improvement rate of lenzilumab group was 91.7% compared with the control group (81.5%), and the inflammatory markers were reduced (Temesgen et al., 2020).

### Inhibition of Janus Kinase Pathway

Janus kinase signal transduction and transcription activator (JAK-STAT) signaling pathway can regulate cell growth, differentiation, survival, and pathogen resistance (Owen et al., 2019). In addition, a large number of cytokines involved in the inflammatory response and autoimmune diseases transduce signals through JAK-STATs (Banerjee et al., 2017).

The JAK1/2 inhibitor ruxolitinib is a kind of drug for the treatment of autoimmune diseases, which can reduce inflammatory cytokines. A clinical study of 601 patients in Italy and Spain showed that the JAK inhibitor neratinib inhibited the release of pro-inflammatory cytokines and protected organs with high expression of ACE2 receptors. The mortality rate and the need for invasive mechanical ventilation in the neratinib group were 16.9%, about half of those in the untreated group (34.9%) (Stebbing et al., 2021). Therefore, blocking the JAK/STAT pathway has potential value in the treatment of hyperimmune syndrome in COVID-19 (Bagca et al., 2020).

### Corticosteroids

Corticosteroids are widely used in inflammatory diseases and ARDS (Barnes et al., 2011) to calm the CS (Pearson et al., 2021) because of their anti-inflammatory and immunosuppressive effects (Martin-Loeches et al., 2021). However, corticosteroids may cause increased SARS-CoV-2 viral load and prolonged infection, even leading to adverse reactions such as liver injury and multiple organ failure, because corticosteroids inhibit immune surveillance (Li et al., 2021). Corticosteroids should not be used to treat lung injury or shock caused by 2019-nCoV (Russell et al., 2020). Thus, in the course of the treatment, corticosteroids have produced controversial and inconclusive results (Lupia et al., 2020; D'Elia et al., 2013; Simmons and Farrar, 2008). From July to September 2020, many clinical trial reports with a positive effect based on the use of hormone were published. For example, dexamethasone can reduce 28-days mortality in patients who require mechanical ventilation (Horby et al., 2020; Olender et al., 2020; Tomazini et al., 2020). World Health Organization organized a meta-analysis of seven clinical trials worldwide, involving 1703 critically ill patients who were treated with corticosteroids including dexamethasone (low dose 6 mg/d, high dose 20 mg/d), hydrocortisone (200 mg/d), and methylprednisolone (80 mg/d) for 7–10 days. The studies showed a 20% reduction in 28-days all-cause mortality. And there was no evidence of an increased risk of serious adverse events (Sterne et al., 2020).

Based on such studies, WHO has published guidelines about corticosteroids for the treatment of patients with COVID-19. In these guidelines, the panel recommended systemic corticosteroid therapy for critical patients with COVID-19. However, there are no clear, objective and quantifiable indicators. Therefore, the “use boundary” of hormonal agents is an urgent clinical issue to be addressed in COVID-19 treatment. Keller et al. used the initial level of CRP >/= 20 mg/dl as the standard for the use of glucocorticoids (Keller et al., 2020). Recently, Cai j et al. Studied 12,862 cases of COVID-19 and found that low-dose corticosteroid therapy can reduce 60-days mortality in patients with neutrophil to lymphocyte ratio greater than 6.11 (Cai et al., 2021). To assess the safety of glucocorticoids, Xian Wang et al. found no significant difference in the incidence of serious adverse events in a meta-analysis of 6,250 patients compared with patients who did not use glucocorticoids (Ma et al., 2021).

### Convalescent Plasma Therapy

The majority of patients who recovered from COVID-19 develop antibodies (Malani et al., 2020). The affinity of the SARS-CoV-2 antibody is correlated with higher neutralization titer (Benner et al., 2020). And the safety of convalescent plasma therapy has been initially evaluated (Joyner et al., 2020b; Joyner et al., 2020c). Infusion of it into the patients’ body can effectively enhance the humoral immune response, reduce the viral load, and inhibit the potential cytokine response (Hung et al., 2011; Shen et al., 2020; Xi et al., 2020). Kai D et al. used convalescent plasma with antibody titer ≥1 ∶ 640 to treat COVID-19 patients, clinical indicators were significantly improved, including an increase of plasma antibody titer, lymphocyte number, a decrease of CRP, viral load, and good tolerance (Duan et al., 2020).

Early randomized trials using convalescent plasma therapy focused on hospitalized patients with moderate or severe disease severity proved that the treatment effect was not obvious (Agarwal et al., 2020; Li et al., 2020; Simonovich et al., 2020). On February 18, 2021, listser et al. conducted a randomized controlled trial of 160 elderly patients who presented with mild symptoms within 72 h. This study compared 250 ml of convalescence-stage plasma with anti-SARS-CoV-2 spines (S) protein IgG titer >1∶1,000 with a normal saline placebo. Finally, they found recovery period plasma slowed down the progression of disease (Libster et al., 2021). Four retrospective studies found that high-titer antibody plasma can reduce mortality within 72 h after infection with the SARS-CoV-2 virus (Joyner et al., 2020a; Joyner et al., 2021; Klassen et al., 2021; Klassen et al., 2021). Therefore, COVID-19 convalescent serum should be used before the onset of early symptoms or autohumoral response (Gharbharan et al., 2021).

### Mesenchymal Stem Cells Therapy

Mesenchymal stem cells (MSC) can be obtained from bone marrow, organ stroma, and other connective tissues (Naji et al., 2019). MSC can interfere with the activation and maturation of antigen-presenting cells, inhibit the proliferation of T lymphocytes, and affect the differentiation of dendritic cells, thereby playing a role in immune regulation and immunosuppression (Duffy et al., 2011). Furthermore, MSC can sense inflammatory cytokine signals and migrate to the inflammatory site, to play the role of inflammatory chemotaxis (Wang et al., 2014). Importantly, MSC has low immunogenicity, so there is less chance of immune rejection (Ankrum et al., 2014). MSC treatment of COVID-19 is safe and well-tolerated, and has shown good efficacy in shortening the course of the disease, reducing lung injury, and lowering cytokine levels (Meng et al., 2020; Yip et al., 2020; Häberle et al., 2021; Shi et al., 2021). The underlying mechanism of MSC in the treatment of COVID-19 is not elucidated, but current clinical evidence suggests that MSC-based therapy has the potential to be a supporting strategy (Zhu, et al., 2020).

### Other Treatments

Because of the immunopathological characteristics of COVID-19, there are many potential COVID-19 immunotherapy strategies. For example, IL-15 immunotherapy (Kandikattu, et al., 2020) and anti-TNFα antibodies (Kunisaki, et al., 2020) are also promising therapeutic strategies. In addition, NK cell-based therapy helps protect the body from SARS-COV-2 infection and enhances the immune response (Market, et al., 2020). Treg cell-based therapy, by overcoming immune dysfunction, will also be an effective treatment for COVID-19 (Yang, et al., 2020).

## Discussion

In this review, several commonly used cytokine inhibitors were summarized, including inhibition of IL-6 signaling, IL-1 signaling, GMC-SF signaling, and JAK pathway. These cytokine inhibitors have therapeutic benefits for patients with COVID-19 but there are still some limitations. First, not all regions have the conditions to detect the level of cytokine storms in the circulatory system. Second, the levels of cytokines in the circulatory system may not accurately reflect the levels in local tissues. Third, cytokines are an important part of the antimicrobial response in the body, and patients with CS have immunodeficiencies, so the use of cytokines targeting inhibitors may lead to a reduced ability to eliminate SARS-CoV-2 (Lauder et al., 2013). To prevent secondary infection, it can be combined with antiviral drugs (such as oseltamivir) and broad-spectrum antibiotics (Wong et al., 2017).

At present, the application of corticosteroids in viral pneumonia is also controversial, because it can not only reduce the excessive inflammatory response but also delay virus clearance due to immunosuppressive effects. High-dose glucocorticoid therapy for COVID-19 does carry the risk of secondary infection and long-term complications. However, for severe patients, multiple organ damage caused by a large number of inflammatory factors may speed up the exacerbation of the disease and even threaten life. Therefore, when it is used in severe patients with COVID-19, the timing, dosage, and course of treatment should be precisely controlled. Based on the current research status, advantages and disadvantages should be balanced carefully before using corticosteroids. The dose of corticosteroids should be low to moderate (Shang et al., 2020).

There is increasing evidence that convalescent plasma therapy may be more effective in preventing diseases in contact than in treating diseases (Casadevall et al., 2020). Since the virus replicates very rapidly in the patient’s body, the optimal time for plasma therapy is likely to be missed. In the future, the optimal dose and timing, as well as the definite clinical benefit of CP therapy, need to be further investigated in randomized clinical studies.

In previous studies, ACE2 was universally acknowledged as the COVID-19 receptor protein. However, Chinese scientists recently conducted the largest single-cell study in the world, sequencing and data analysis of nearly 1.5 million single-cell transcriptomes (Ren et al., 2021). In this study, the nucleic acid sequence of COVID-19 was detected in a variety of immune cells. However, ACE2 was hardly expressed in immune cells. Therefore, we can speculate that COVID-19 may have potential new receptors, a wider host cell range, and even more transmission channels. Considering that the host’s local or systemic response mechanism to infection has not yet been fully clarified, more researches are needed to clarify the precise pathological mechanism. It is believed that in the future, more immune modulators will be used for the treatment of cytokine storm-related diseases.
